# Development of a System to Deliver Inhalational Antibiotics to Marmosets

**DOI:** 10.3390/antibiotics14060554

**Published:** 2025-05-29

**Authors:** Rachel E. Ireland, Stuart J. Armstrong, Carwyn Davies, James D. Blanchard, Francis Dayton, Igor Gonda, Sarah V. Harding, Michelle Nelson

**Affiliations:** 1 Defence Science and Technology Laboratory (Dstl), Porton Down, Salisbury SP4 0JQ, UK; sjarmstrong@dstl.gov.uk (S.J.A.); cdavies@dstl.gov.uk (C.D.); svharding@dstl.gov.uk (S.V.H.); mnelson@dstl.gov.uk (M.N.); 2 Aradigm Corporation, Hayward, CA 94545, USA; sweeneytds@gmail.com (J.D.B.); francisdayton@outlook.com (F.D.); igonda@respidex.com (I.G.)

**Keywords:** liposomal encapsulated ciprofloxacin, nebulisers, non-human primate model, pharmacokinetics

## Abstract

Background: Inhalational antibiotics have been used effectively to treat chronic diseases such as *Pseudomonas aeruginosa* infections associated with cystic fibrosis. This approach may enhance treatment options for difficult-to-treat, acute pneumonic diseases. Liposomal encapsulated ciprofloxacin (Lipoquin and/or Apulmiq) has provided protection in murine models of plague, anthrax, Q fever and tularemia. Development of the ability to deliver these drugs to nonhuman primates (NHPs) would enable further extrapolation of the data observed in small animal models of infection to humans. Methods: In this study, the methodology was established to deliver Apulmiq to common marmosets (*Callithrix jacchus*). Marmosets were anaesthetised with a novel, reversible anaesthetic comprising fentanyl, medetomidine and midazolam (FMM). They were placed into plethysmography tubes with their heads in an exposure chamber. The LC Sprint jet nebuliser or Pari eFlow Rapid nebuliser were used to aerosolise Apulmiq into the exposure chamber. Animals were euthanised after dosing and the concentration of ciprofloxacin was assessed in the plasma and lungs of the animals. Results: Non-compartmental pharmacokinetic analysis determined that a 30 min exposure of drug was required to reach a human-equivalent target dose of 0.8 mg/kg body weight in the lungs. Conclusions: This approach can now be used to assess the efficacy of inhalational liposomal ciprofloxacin in NHP infection models.

## 1. Introduction

Inhaled antibiotics, specifically azetreonam, colistin, levofloxacin and tobramycin, have been effectively used for the treatment of chronic diseases such as *Pseudomonas aeruginosa* infections associated with cystic fibrosis [1]. More recently, inhaled liposomal amikacin has been approved for the treatment of *Mycobacterium avium* complex respiratory infections [2]. Their use for the treatment of acute infections in humans is relatively unexplored even though there are a number of advantages of such formulations, including the potential to reduce the risk of antimicrobial resistance developing due to the delivery of the antibiotic targeted to the site of infection [3]. Two forms of liposomal encapsulated ciprofloxacin, Lipoquin^®^ and Apulmiq^®^ (formerly known as Pulmaquin^®^), have been used to treat acute lung infections in animal studies [4,5]. These liposomal encapsulated formulations were developed by Aradigm Corporation. The ability of encapsulation to reduce the systemic exposure of ciprofloxacin [6,7] may become more important following recent guidelines from the Medicines and Healthcare Products Regulatory Agency regarding the future use of fluoroquinolones [8]. Their recommendation to restrict the use of fluoroquinolones due to the risk of potential side effects may fundamentally change clinical practice.

The delivery of antibiotics to the lungs is dependent on appropriate delivery devices. Three types of devices are typically used: nebulisers, dry particle inhalers and meter-dose inhalers [9]. These devices have their advantages and disadvantages, although inhalational antibiotics are most commonly delivered via nebulisation due to the higher effective doses required for antibiotics [10,11]. Nebulisation can be achieved using jet nebulisers, ultrasonic nebulisers or vibrating mesh nebulisers. Jet and/or vibrating mesh nebulisers have been utilised in humans to deliver tobramycin, azetreonam, levofloxacin, amikacin, colistin and liposomal ciprofloxacin [2,10,11,12,13].

Both Lipoquin and Apulmiq have been investigated in human clinical trials for non-cystic fibrosis bronchiectasis and chronic lung infections with *P. aeruginosa* using a Pari LC Sprint^®^ jet nebuliser [14]. In the ORBIT-3 and ORBIT 4 trials, the nebuliser was loaded with 189 mg of Apulmiq. Data from Aradigm indicated that ~50% of this concentration is nebulised, with ~60% of that in a size range that reaches the human lung. Therefore, ~30% of the concentration of drug loaded into the nebuliser reaches the lung (i.e., 56.7 mg) so for a 70 kg human the dose is 0.8 mg/kg body weight. Apulmiq was assessed in these trials, and it is the most developed of the two formulations. An extensive human safety database exists, and manufacturing facilities for the drug product on a commercial scale under Good Manufacturing Practice (GMP) are available. Apulmiq is a 1:1 mixture of liposomal ciprofloxacin (Lipoquin, 50 mg/mL) and free ciprofloxacin (20 mg/mL). This gives the immediate effect of the free ciprofloxacin combined with a prolonged, sustained delivery of ciprofloxacin from the encapsulated component. Due to the advanced nature of Apulmiq, NHP data is required to further extrapolate the data observed in small animal models of pathogens of defence interest to humans. Several infection models that result in acute pneumonia have been established in the small New World monkey, the common marmoset (*Callithrix jacchus*) [15,16]. The aim of this work was to develop the methodology to deliver aerosolised Apulmiq to marmosets in order to establish the pharmacokinetics prior to assessing this drug in infection models.

## 2. Results

### 2.1. Establishment of the Marmoset Inhalational Therapy System (MITS)

**Figure 1:**
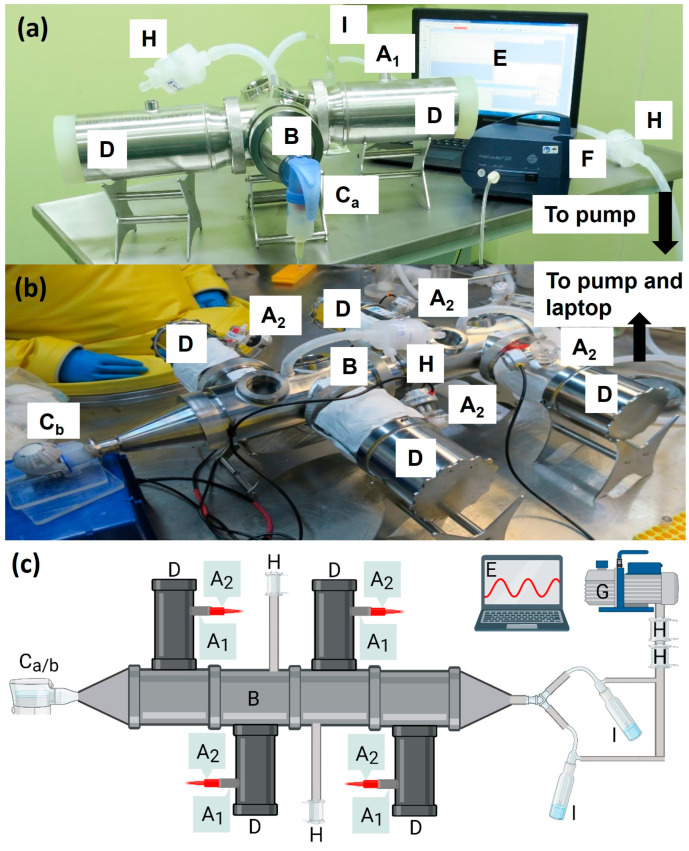
The marmoset inhalational therapy system (MITS) set up to enable delivery of aerosolised Apulmiq to marmosets (**a**) outside of containment (**b**) or within a high containment isolator system. (**c**) A schematic of the system used in high containment (created with BioRender.com). Animals were anaesthetised and placed into the plethysmography tubes (D) with their heads in the exposure chamber (B). The drug was aerosolised using either the Pari LC Sprint^®^ jet nebuliser (C_a_) or the Pari eFlow Rapid nebuliser system (C_b_). For the LC Sprint, the Pari Boy SX compressor (F) generated the airflow to nebulise the drug at a rate of 5.1 L/min. The vacuum pump (G) drew the nebulised drug through the chamber at a rate of 5.9 L/min. Pall Emflon filters ii in Kleenpak capsules (KA1V002PV2G) (H) were used to filter air leaving the system and to maintain a steady pressure in the exposure unit. Impingers (I) were placed downstream of the exposure unit to sample the air to estimate the concentration of antibiotic present in the aerosol. A pneumotachograph (A_2_) was attached at the port (A_1_) to monitor the volume inhaled by the individual animal using plethysmography in real time, which was visualised on the laptop (E).

A system was developed to deliver aerosolised Apulmiq to marmosets (Figure 1). The system comprised a mechanism to anesthetise the animals for the administration of the drug, a nebuliser to generate the aerosolised drug, a vacuum pump to gently pull the aerosolised drugs through the system and a central exposure chamber to which tubes containing the animals were connected. Anaesthetised animals were placed into a bespoke plethysmography tube (EMMS, Bordon, UK) and a Fleisch pneumotachograph (EMMS, UK) was attached to monitor the real-time breathing rate of animals during exposure to the antibiotic. Up to four plethysmography tubes were attached to a central exposure chamber which had a nebuliser attached to one end and a vacuum pump at the other in order to create a dynamic airflow of aerosolised antibiotic. Initially, a Pari LC Sprint^®^ jet nebuliser (Midlothian, IL, USA), powered by the Pari Boy SX compressor, was used to aerosolise the antibiotic, although this was later replaced by the Pari eFlow Rapid nebuliser.

### 2.2. Establishing a Novel Anaesthetic Protocol for Marmosets

A novel, reversible anaesthetic protocol was established to ensure animals were anaesthetised for a sufficient duration (up to 60 min) on repeated occasions, with minimal impact on their well-being. A cocktail of fentanyl, medetomidine and midazolam (FMM) was investigated, based on work performed in rhesus macaques [17]. Initially, animals were anaesthetised and exposed to Apulmiq in a stepwise manner to assess the response to and recovery from the FMM cocktail. The onset to sedation was between 5 and 10 min (typically around 8 min), although this was more effective in a quiet environment with low light.

Following 15 to 60 min of inhalational dosing, the sedation was reversed with a combination of naloxone, atipamezole and flumazenil, and the animals were directly observed for 1 h. Within 6 to 8 min, animals were responsive to stimuli, fully mobile, co-ordinated and displaying normal behaviours. All animals were eager to eat and drink from between 5 and 30 min post-reversal. All animals recovered and returned to their normal behaviour much faster than using the standard anaesthetic ketamine (typically 3 to 4 h).

### 2.3. Characterisation of Aerosolised Apulmiq in the Lungs of Marmosets

**Figure 2:**
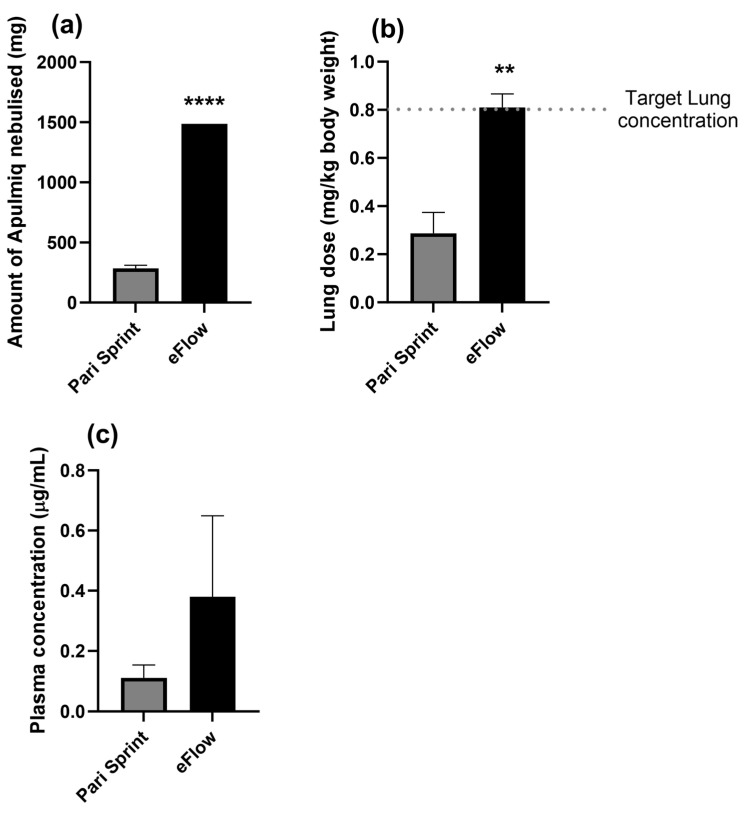
Assessment of the delivery of aerosolised Apulmiq to marmosets. Two (Pari eFlow Rapid) and three (LC Sprint Star jet nebuliser) animals, were exposed to Apulmiq for 60 min, and the concentration of Apulmiq aerosolised (**a**), the lung dose received (**b**) and the plasma concentration (**c**), was compared for each condition. An unpaired *t*-test was performed where ** *p* = 0.0053 and **** *p* < 0.0001.

In order to optimise the dose of Apulmiq delivered to the animals, lungs and blood were collected from the same animals described above at 1 h post-exposure. To align with human data, the target lung dose was 0.8 mg/kg body weight of Apulmiq. Initially, three individual animals were exposed to aerosolised Apulmiq using the LC Sprint jet nebuliser for 60 min and, during this period, a mean of 286 mg of Apulmiq was nebulised (Figure 2a). The mean concentration of ciprofloxacin in the lungs when the animals were euthanised immediately 1 h later was 0.29 mg/kg body weight (Figure 2b), and the mean plasma concentration was 0.11 µg/mL (Figure 2c). This was not sufficient to successfully deliver the target lung dose of 0.8 mg/kg body weight. Rather than increasing the time animals needed to be anesthetised to deliver the required concentration of drug, a more efficient, alternative nebuliser, the Pari eFlow Rapid nebuliser was used to deliver Apulmiq to two further animals for 60 min. There was a significant increase in the total amount of drug nebulised (1485 mg compared to 271 mg using the Pari LC Sprint jet nebuliser; *p* < 0.0001). This resulted in an increase in the concentration of ciprofloxacin detectable in the lungs to 0.85 mg/kg body weight for one animal or 0.77 mg/kg body weight for the other animal. The concentrations of ciprofloxacin detected in the plasma were 0.19 μg/mL and 0.57 μg/mL, respectively, for each animal. Therefore, the use of the Pari eFlow Rapid nebuliser to deliver Apulmiq for 60 min was determined to be the appropriate regimen to use for a more extensive pharmacokinetic assessment of the drug.

In addition, the particle size distribution of Apulmiq and empty liposomes (liposomes formed without the addition of ciprofloxacin) generated by the eFlow Rapid nebuliser was assessed. Both formulations gave a mass median aerodynamic diameter (MMAD) of between 2 μm and 3 μm (2.13 μm and 2.89 μm for Apulmiq and empty liposomes, respectively), with a geometric standard deviation (GSD) of 1.49 and 1.72, respectively. This suggests that the aerosol droplets containing aerosolised antibiotic could reach the deep lung of the animals (bronchioles and alveoli).

### 2.4. Pharmacokinetics of Apulmiq in the Plasma and Lungs of Marmosets

**Figure 3:**
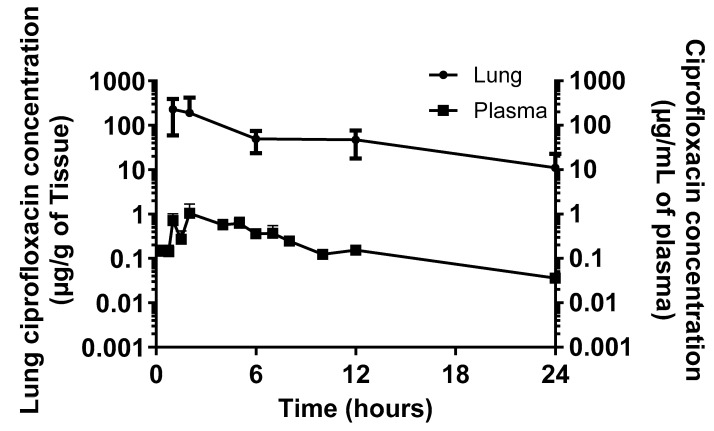
The time–concentration profile of ciprofloxacin in the lungs or the plasma of marmosets following aerosolisation of Apulmiq. Marmosets were exposed to Apulmiq for 1 h and the concentration of ciprofloxacin determined using LC-MS.

The concentration of ciprofloxacin was determined in the plasma and lungs from a cohort of 15 marmosets administered a single dose of Apulmiq for 1 h (Figure 3). The mean dose of antibiotic received in the lung was 1.94 mg/kg body weight due to a higher breathing rate in this cohort of animals. Non-compartmental PK analysis of the mean concentration–time profiles of ciprofloxacin in the lung and plasma was performed using WinNonlin Phoenix v.6.1 (Pharsight Corp., Sunnyvale, CA, USA) (Table 1). A maximum concentration (C_max_) of 226.5 μg/g was reached by 1 h (T_max_) in the lungs of animals. In the plasma, the C_max_ of 1.05 μg/mL was achieved at a T_max_ of 2 h post-dosing. The terminal half-life (T_1/2_) of Apulmiq was 5.6 and 6.03 h in the lungs and plasma, respectively. The area under the concentration–time curve (AUC) was 1654 μg·h/mL and 6.20 μg·h/mL in the lungs and plasma, respectively.

Compartmental analysis of the 60 min Apulmiq exposure data from the marmoset was completed and the model fit to the data used to simulate different exposure durations, enabling alignment with the pharmacokinetic parameters of a dose used in a murine model that was efficacious [18]. These simulations indicated that a 30 min dosing duration in marmosets would result in comparable lung concentrations to the simulated mouse values (Table 1). The target lung dose of 0.8 mg/kg body weight was achieved and an AUC and C_max_ of 682 μg·h/mL and 93.4 μg/mL were predicted in the lungs of marmosets, which compared to 710 μg·h/mL and 100.8 μg/mL in the mouse. The plasma values were also comparable with a predicted AUC and C_max_ of 2.51 μg·h/mL and 0.43 μg/mL in the marmoset compared to 1.76 μg·h/mL and 0.40 μg/mL in the mouse. Pharmacokinetic data are not available from the lungs of humans, although the AUC and C_max_ in the plasma were 2.034 ± 1.895 μg·h/mL and 0.195 μg/mL, respectively.

### 2.5. Phenotype Characterisation of Alveolar Macrophages in the Lungs of Marmosets Following Aerosolisation of Apulmiq

**Figure 4:**
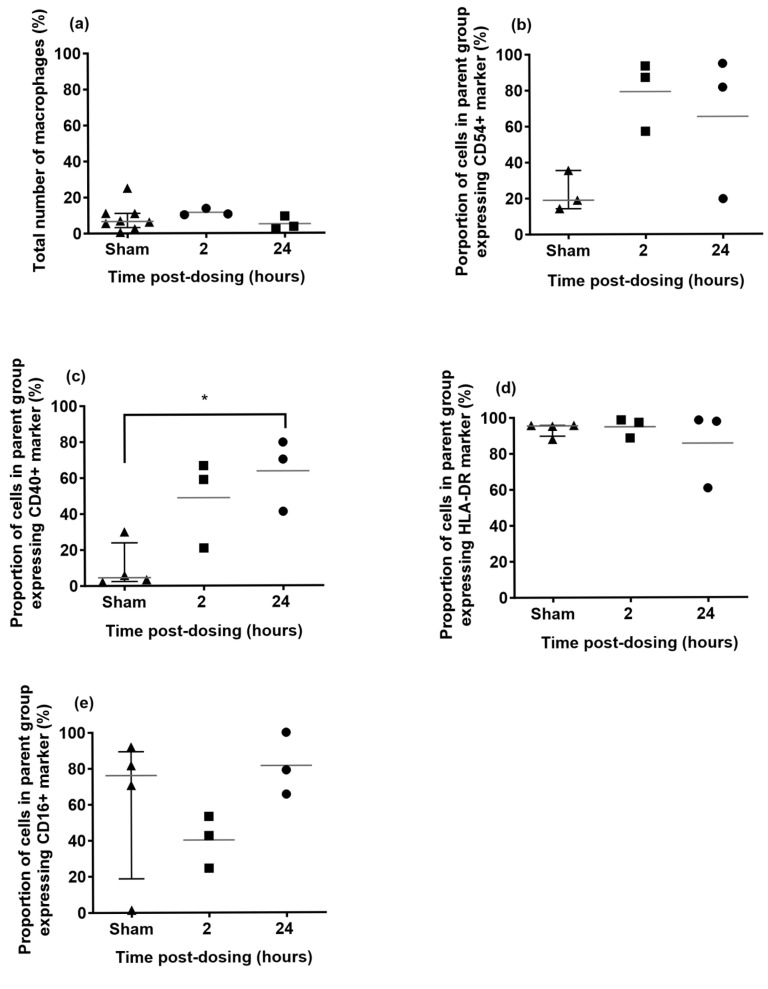
Phenotyping of alveolar macrophages from marmosets exposed to Apulmiq or a sham, phosphate buffered saline (PBS). Animals exposed to Apulmiq were euthanised 2 or 24 h later. (**a**) Total number of macrophages, (**b**) expression of the migratory marker, CD54^+^, (**c**) expression of the inflammatory marker, CD40^+^, (**d**) expression of the MHC class II marker, HLA-DR^+^ and (**e**) expression of the alternative activation marker, CD16^+^. A one-way ANOVA was performed, where * *p* = 0.0190.

The number of macrophages in the lung did not increase at 2 h and at 24 h post-dosing (Figure 4). This was comparable to historical data generated at Dstl when marmosets were exposed to aerosolised PBS. However, there was upregulation in the expression of the migratory marker, CD54^+^ and the inflammatory marker, CD40^+^ on the macrophages at both 2 h and 24 h post-dosing. There was a transient reduction in the expression of the alternative activation marker, CD16^+^ on the macrophages at 2 h post-dosing, but minimal changes to the expression of the HLA-DR marker at either time. These differences were not apparent for the blood monocytes, with only the expression of CD40^+^ observed to increase. 

## 3. Discussion

The administration of antibiotics by the inhalational route may offer advantages over the administration of antibiotics by the oral route, including the achievement of high concentrations in the lungs while minimising systemic exposure to the antibiotic, which reduces the potential for the development of antimicrobial resistance, and systemic adverse effects [19,20]. The overall aim of this work was to develop a system to deliver antibiotics by the inhalational route to a non-human primate model, with the additional aim of assessing the pharmacokinetics of Apulmiq, prior to undertaking efficacy studies.

The purpose of this work was to develop methodology to deliver inhalational antibiotics to marmosets. Previous experience with mice indicated that it could take up to 60 min to administer the required dose of antibiotic by the inhalational route [5,18]. Delivery would also need to be performed at least once a day for up to 7 days, and the marmosets would need to be anesthetised for inhalational delivery. Therefore, the routine anaesthetic regimen used at Dstl for marmoset studies (ketamine with or without medetomidine) was not considered appropriate. Ketamine is a dissociative anaesthetic commonly used in nonhuman primates, which is extremely effective and has a high safety margin. The onset of sedation is quick, occurring within 1 to 2 min. However, recovery from ketamine is slow, taking a minimum of 20 min before the animal is responsive, but it can take up to 4 h before the animals return to normal eating and drinking behaviours (personal observations). Fentanyl, midazolam and medetomidine (FMM) are all reversible anaesthetics that have been previously used effectively in combination in the rhesus macaque [17]. The concentrations of these drugs were adjusted for use in marmosets based on the experience of the named veterinary surgeon (NVS) at Dstl. Unlike the protocol established for rhesus macaques, flumazenil was used to reverse midazolam, in conjunction with the use of atipamezole and naloxone to reverse medetomidine and fentanyl, respectively. This regimen provided the appropriate amount of sedation for the required duration (up to 60 min) and had a minimal impact on the animals.

The equipment used to deliver the antibiotic to marmosets was a simplified adaptation of the equipment used to deliver aerosolised pathogen to animals and was in line with the system used to deliver inhalational antibiotics to mice [5,18,21]. The anaesthetised animals were placed within bespoke plethysmography tubes and placed with their head into the central exposure manifold. A similar head-only approach has been reported for delivery of aerosolised remdesivir to African green monkeys (AGM), although those animals were conscious and chair-restrained with their head placed in a head-dome [22]. Alternative approaches to delivering aerosolised compounds to NHPs include the use of a nasal cannula or face mask [23,24]. Each approach has advantages and disadvantages. The use of a nasal cannula or face mask will result in directed delivery to the respiratory tract of the animal. One downside of a nasal cannula is that the nose filters out particles which is not observed with mouth breathing. Changing the port of entry to the face mask changed the deposition profile of the compound [24]. For head only exposure, larger amounts of antibiotic are required to be nebulised, as only a small proportion of the drug will be inhaled. The use of light anaesthesia is known to reduce the breathing rate of animals, so the inhaled dose in the animals will also be reduced or require a longer duration of exposure to reach the target concentration. However, this reduces the safety hazard when handling animals infected with pathogens. Not using light anaesthesia may increase the stress of the procedure, changing the natural breathing pattern and result in a reduction in the welfare of the animal.

In addition, the type of device used to generate the aerosolised antibiotic will affect the concentration of the drug that can be delivered to the lungs. In these studies, replacing the Pari LC Sprint jet nebuliser with the Pari eFlow Rapid nebuliser system (using a vibrating mesh) increased the amount of drug nebulised by just over five-fold in a 60 min window (from 293 to 1485 mg). This resulted in over a three-fold increase in lung dose (from 0.25 to 0.81 mg/kg). The shorter nebulisation time for the Pari eFlow Rapid nebuliser had previously been reported when compared to other Pari jet nebulisers [25,26,27]. The MMAD of aerosolised Apulmiq in these studies was 1.84 μm, which would theoretically result in approximately 5% of them depositing in the oropharynx and approximately 40% in the alveoli [27]. Despite the difference in particle size, the deposition of particles in both humans and NHPs were similar when generated using vibrating mesh nebulisers (such as the Pari eFlow Rapid) and jet nebulisers [23,28].

The pharmacokinetic profile of a single inhalational dose of Apulmiq was determined following a 60 min exposure to the drug in a cohort of marmosets. Following this regimen, a lung dose of 1.94 mg/kg body weight was determined, which is significantly higher than the target lung dose of 0.8 mg/kg body weight. Compartmental analysis was performed on these data to align the pharmacokinetic parameters with an efficacious dose used in murine models of plague and tularemia [7,18]. This resulted in a reduction in the required duration of exposure to 30 min, thereby improving animal welfare. The resulting AUC of 682 μg·h/mL in the lungs was slightly lower than the AUC in the Balb/c mouse of 710 μg·h/mL [18]. The AUC was used as the comparative metric as the AUC/MIC ratio is considered a specific pharmacokinetic/pharmacodynamic (PK/PD) target for ciprofloxacin [29]. The comparison of the AUC in the lungs to humans is not possible as the collection of lung tissue is not assessed in clinical trials. Occasional bronchiolar lavage samples could be collected in humans, although it is challenging to do this on multiple occasions in a single patient. Alternatively, it is possible to compare the pharmacokinetic parameters in the plasma between species. The 30 min dosing regimen simulated for marmosets predicted an AUC of 2.51 μg·h/mL. This prediction is within the range observed in humans (2.03 ± 1.90 μg·h/mL), although it is slightly higher than the AUC obtained with the effective dosing regimen used in Balb/c mice (1.76 μg·h/mL). As anticipated, the half-life of liposomal encapsulated antibiotics is greater than orally administered ciprofloxacin in the marmoset: 6.03 h for inhalational delivery of Apulmiq compared to 1.9 h following oral administration of ciprofloxacin [30]. This is also observed in humans, where the half-life of Apulmiq is approximately 9 h compared with 3 to 5 h for orally administered ciprofloxacin [31]. This phenomenon is not unique to ciprofloxacin, as encapsulation of gentamicin within liposomes increases the half-life in mice from 1 h (free gentamicin) to 3.8 h [32].

Marmoset alveolar macrophages were shown to be activated following inhalational delivery of Apulmiq. High concentrations of ciprofloxacin can have immunomodulatory effects, although the concentrations required are above those achieved in vivo [33]. Liposome encapsulated ciprofloxacin was shown to increase the phagocytic activity of macrophages and enhanced the intracellular killing of *Staphylococcus aureus* [34]. Therefore, the activation of the alveolar macrophages in this study may be beneficial and will require further work to explore.

In conclusion, a system has been developed to deliver inhalational antibiotic—a mixture of free and liposomal encapsulated ciprofloxacin (Apulmiq) to marmosets, and a target human-equivalent dose of 0.8 mg/kg body weight of Apulmiq was achievable in the lungs, which will enable the efficacy of future studies.

## 4. Materials and Methods

### 4.1. Animals

Common marmosets (*C. jacchus*) were obtained from the Dstl Porton Down breeding colony (Salisbury, UK) as healthy, sexually mature animals, aged between 36 and 60 months (weighing between 358 and 565 g). They were housed as female/vasectomised male pairs and were given species-appropriate environmental enrichment such as access to forage mix containing preferred food items, sleeping boxes and puzzle feeders. All animals were acclimatised for a minimum of 7 days in the experimental room prior to the start of the study.

### 4.2. Administration of Sedation

Prior to the inhalational exposure to Apulmiq, marmosets were sedated with a novel cocktail of fentanyl (0.01 mg/kg, Hameln Pharma, Gloucester, UK) medetomidine (0.06 mg/kg, Orion Pharma, Reading, UK)) and midazolam (0.5 mg/kg, Hameln Pharma) by the intramuscular (i.m.) route. Post-antibiotic exposure, the cocktail was reversed using a combination of naloxone (0.01 mg/kg, Hameln Pharma), atipamezole (0.3 mg/kg, Orion Pharma) and flumazenil (0.01 mg/kg, Hameln Pharma) administered by the i.m. route.

### 4.3. Inhalational Delivery of Antibiotics

The marmoset inhalational therapy system (MITS) was used to expose animals to aerosolised Apulmiq for a defined period of time, either 16, 33 or 60 min. An Aerodynamic Particle Sizer (APS) 3321 and Aerosol diluter 3302A (TSI, Buckinghamshire, UK) were used to determine the particle size distribution. The total dose of nebulised Apulmiq per exposure was calculated as (total input volume—total volume remaining at end of exposure) × concentration of Apulmiq (35 mg/mL).

### 4.4. Antibiotic

Apulmiq (Aradigm Corporation, Hayward, CA, USA) was produced by mixing equal volumes of Lipoquin (liposomal encapsulated ciprofloxacin) (50 mg/mL) and free ciprofloxacin solution (20 mg/mL) to give a final concentration of 35 mg/mL. The concentration of ciprofloxacin was determined in the lungs and plasma from each animal by liquid chromatography–mass spectrometry (LC-MS). Samples were prepared as follows: for plasma, 50 μL of plasma standard or sample was mixed with 150 μL of internal standard in acetonitrile (MeCN), centrifuged and the supernatant decanted into a clean tube. The supernatants were reduced in volume using a Genevac centrifugal evaporator (Suffolk, UK, 50 min at 40 °C) to approximately 50 μL before injecting into the LC-MS system (Agilent 1100, Santa Clara, CA, USA, CTC PAL, Sciex 3000). The lung sections were weighed, 1 or 2× the volume of 0.1% formic acid added (assuming the lung tissue was 1 g/1 mL) and the tissue homogenised in a Precellys bead homogeniser (Montigny-le-Bretonneux, France, 7 mL tubes containing 2.8 mm beads) using 4 × 20 s cycles at 5000 rpm. A total of 50 μL of the homogenate was mixed with 150 μL of internal standard and prepared as for plasma. Reference standards were prepared as follows: ciprofloxacin was weighed and dissolved in 0.1% aqueous formic acid to give a 1 mg/mL ciprofloxacin stock solution. The stock solutions were prepared freshly on the day of analysis. Ciprofloxacin-d8 was dissolved in 0.1% FA to give a 1 mg/mL stock solution. This solution was diluted to 1 μg/mL in MeCN to give the working internal standard solution. Ciprofloxacin calibration curves in marmoset plasma were prepared consisting of 10 points from 1 ng/mL to 2000 ng/mL. The lung dose per kg body weight was calculated as concentration of ciprofloxacin in whole lung (mg)/weight of animal (kg).

### 4.5. Determination of the Pharmacokinetics of Apulmiq in the Plasma and Lungs of Marmosets

In order to determine the appropriate amount of time to expose animals to antibiotic to achieve the target dose of 0.8 mg/kg body weight of Apulmiq in the lungs, marmosets were exposed to aerosolised Apulmiq for 60 min. Animals were humanely culled at 1 h post-exposure (n = 5), and blood and lung tissue were collected and the concentration of ciprofloxacin determined. Blood was removed by cardiac puncture into lithium heparin-coated tubes, centrifuged for 5 min at 10,000 rpm to separate the plasma and stored at −80 °C prior to analysis. Lungs were collected into universal tubes and stored at −80 °C prior to analysis.

To determine the pharmacokinetic profile, groups of marmosets (n = 3) were exposed to Apulmiq for 1 h and euthanised at 1, 2, 6, 12 or 24 h post-exposure in a sparse random design. At post-mortem, blood was removed by cardiac puncture into lithium heparin-coated tubes, and lungs were dissected. Plasma and lung tissue were analysed for ciprofloxacin content, and the analysts were blinded to the timepoint the sample was collected. Samples of the blood and lung were also analysed for immunological changes. There was no data exclusion in these studies.

The drug concentration data was modelled using the Phoenix WinNonLin (Pharsight v 6.1) software (Certara, Radnor, PA, USA) to determine parameters’ half-life (t_1/2_), area under the curve (AUC), maximum concentration of drug (C_max_) and time to the maximum concentration (T_max_). Nonparametric superposition modelling was performed to assess the effect of changing the duration of dosing. Compartmental modelling was performed to assess the effect of changing the initial dose and the frequency of dosing.

### 4.6. Immunology

Blood and single-cell suspensions of the lungs were analysed from three animals that were culled at 2 h and 24 h post-exposure (from the pharmacokinetic study) to determine any stimulatory effect of the treatment. Red blood cells (RBCs) were lysed using RBC lysis buffer (BD Biosciences, Oxford, UK). The remaining leukocytes were stained for 40 min at room temperature using a stain mix comprising fluorescently bound anti-human or anti-marmoset antibodies: CD14 (M5E2: 557742) from BD biosciences; MHCII (L243: 307630), CD40 (5C3: 334336), CD16 (3G8: 302018) and CD54 (HCD54: 322716) all from BioLegend (San Diego, CA, USA). The cells were washed with phosphate buffered saline (PBS) and a fixation step in 4% paraformaldehyde (PFA) was carried out for 24 h. Cell phenotypes and activation status were determined by flow cytometry using a BD Canto II FACS machine. Whole cells were detected by nuclear staining, allowing the area of interest to be defined by forward and side scatter. Forward and side scatter were also used to gate areas for the detection of macrophages (M0).

### 4.7. Statistical Analyses

All statistical analyses were performed using GraphPad Prism version 10.0.2. (GraphPad Software, San Diego, CA, USA). Unpaired *t*-tests were used to compare the amount of drug aerosolised using the Pari eFlow Rapid and the LC Sprint Star jet nebuliser and the concentration of antibiotic in the lung and plasma. A one-way ANOVA was performed on the macrophage cell expression over time.

## Figures and Tables

**Table 1:** Pharmacokinetic parameters determined in the plasma and lung homogenates of marmosets exposed to aerosolised Apulmiq.

Species	Lung Dose (mg/kg)	Plasma				Lung Homogenates			
		C_max_ (μg/mL)	T_max_ (h)	AUC (μg·h/mL)	T_1/2_ (h)	C_max_ (μg/mL)	T_max_ (h)	AUC (μg·h/mL)	T_1/2_ (h)
^1^ Marmoset (60 min)	1.94	1.05	2	6.20	6.03	226.5	1	1654	5.6
^2^ Marmoset (30 min)	0.80	0.43	2	2.51	6.03	93.4	1	682	5.6
^3^ Human	0.57	0.195	1.645	2.03 ± 1.90	9.22 ± 1.16	ND	ND	ND	ND
^4^ Mouse (30 min)	0.86	0.40	0.06	1.76	3	100.8	0.02	710	4.9

^1^ Empirical data obtained by exposing animals to aerosolised Apulmiq for 60 min. ^2^ Simulated data using a compartmental model that was fitted to the empirical 60 min exposure data (assuming the relationship between lung dose and length of exposure is linear). ^3^ Expected dose in humans (70 kg) following the nebulisation of 210 g of Apulmiq (Aradigm Corp, unpublished data). ^4^ Empirical murine data generated in a similar manner that was shown to be efficacious in murine models of infection. ND, not determined.

## Data Availability

The original contributions presented in this study are included in the article. Further inquiries can be directed to the corresponding author.

## Crown copyright (2024)

Dstl. This material is licensed under the terms of the Open Government Licence except where otherwise stated. To view this licence, visit http://www.nationalarchives.gov.uk/doc/open-government-licence/version/3 or write to the Information Policy Team, The National Archives, Kew, London TW9 4DU, or email: psi@nationalarchives.gov.uk.
